# Supplementary material for: Tead1a Initiates Transcriptional Priming Through the TEAD1a/YAP‐Notch1‐Spi1/Cebpα Axis to Promote Neutrophil Fate
Source: Adv Sci (Weinh). 2025 Aug 28;12(41):e05441. doi: 10.1002/advs.202505441 (PMC12591127; doi:10.1002/advs.202505441)
Supplement: Supplementary file 4 — Supplementary Dataset [file ADVS-12-e05441-s004.zip › 3-3.zip!/3-3/02-3.cluster/cluster7/cluster7_vlnplot.pdf]

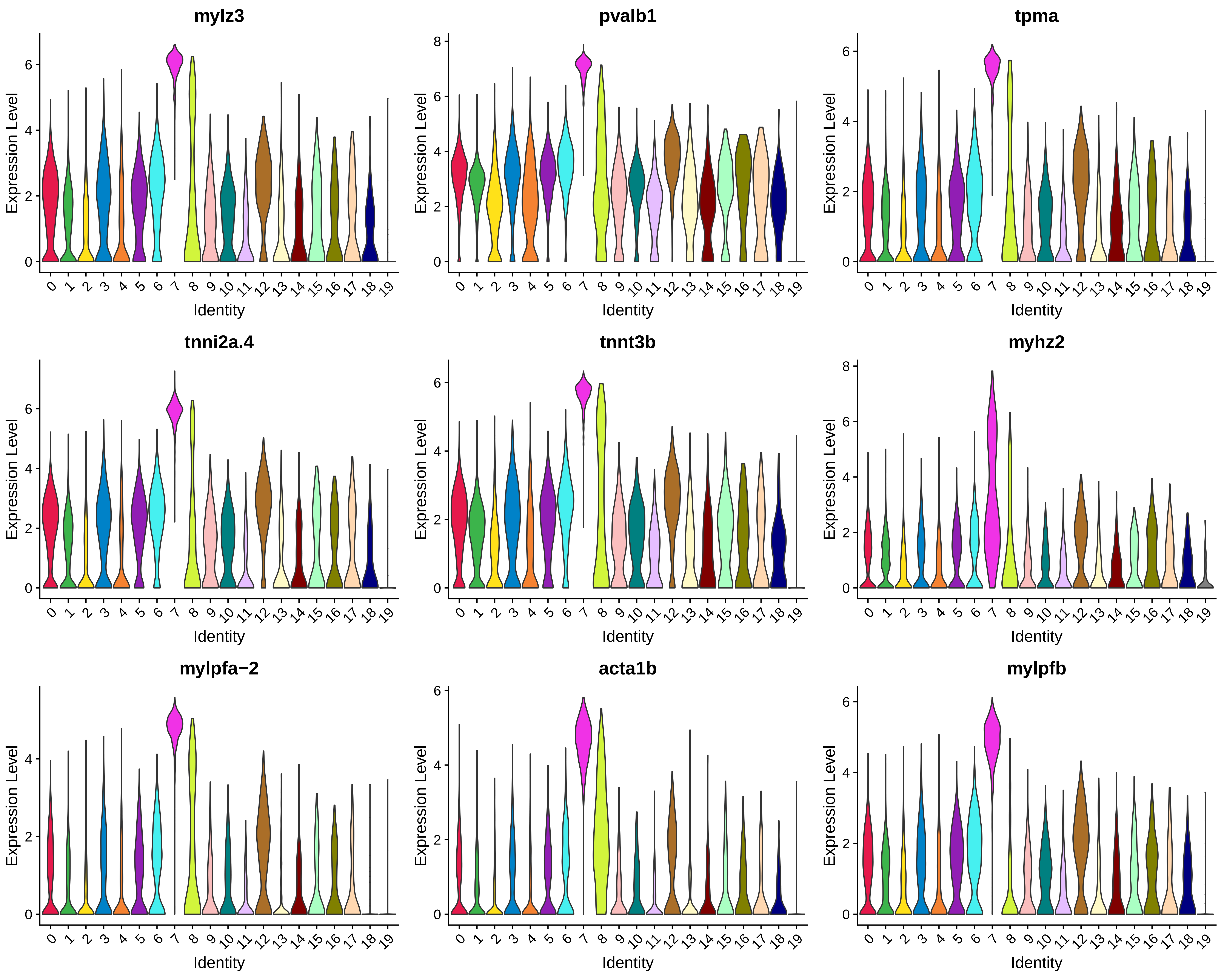
mylz3
pvalb1
tpma
tnni2a.4
tnnt3b
myhz2
mylpfa−2
acta1b
mylpfb
Expression Level
Identity
0 1 2 3 4 5 6 7 8 9 10 11 12 13 14 15 16 17 18 19
